# Laryngeal Manifestation of Forestier’s Disease

**DOI:** 10.3889/oamjms.2016.054

**Published:** 2016-05-10

**Authors:** Jasmina Stojanovic, Sandra Zivanovic, Suncica Sreckovic, Svetlana Jovanovic, Branislav Belic, Sladjana Simovic

**Affiliations:** 1 *Phoniatric Department of ENT Clinic, Clinical Centre Kragujevac, Kragujevac, Serbia*; 2 *Faculty of Medical Science, University of Kragujevac, Kragujevac, Serbia*; 3 *Department of Otorhinolaryngology Health Centre Kragujevac, Kragujevac, Serbia*

**Keywords:** Forestier’s diseaseis, hoarseness, airway disturbances, dysphagia

## Abstract

**BACKGROUND:**

Forestier’s disease is a rare disorder involving bony growths that can occur in various parts of the spinal column, mostly asymptomatic, but these osteophytes, very rarely have been associated with serious complications.

**AIM:**

We report a 69-year-old man who was admitted at foniatric departement for evaluation of presenting hoarseness, dysphagia and laborious breathing.

**CASE PRESENTATION:**

Noninvasive endolaryngeal imaging and radiological examination revealed distortion of left side of the larynx pushing to the right due to bony mass of the anterior part of cervical spine which was prominent at the left side. The symptoms of the patient presented were caused by Forestier’s disease as found by the imiging.

**CONCLUSIONS:**

In clinical practice it is advisable to take into consideration Forestier’s disease as a possible cause of hoarseness and dysphagia in rare cases.

## Introduction

Diffuse idiopathic skeletal hyperostosis (DISH), also known as Forestier’s disease, is a noninflammatory ossification involving at least four contagious vertebral bodies, with intensive formation of osteophytes affecting ligaments, tendons and fascia of the anterior part of the spinal column [1]. It mainly affects males over 50 years of age. The incidence has been estimated at 12% [2] and based on radiological surveys of the cervical spine 2.4-5.4% in older than 40 years of age [3]. Although Forestier’s disease is asymptomatic in general, in the literature were reported dysphagia, dyspnea and dysphonia all together only in rare cases [4].

## Case report

**Figure 1:**
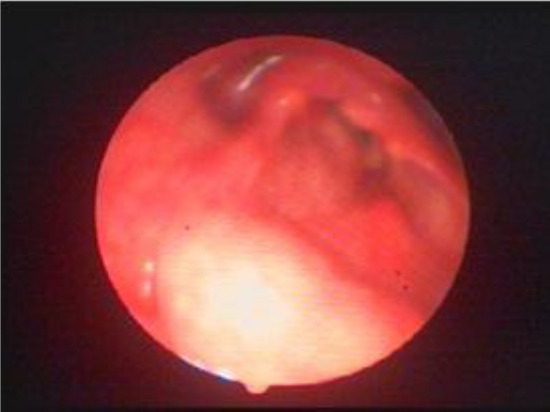
*The first fiberoptic laryngoscopy findings shown distortion of larynx to left*.

A 69-year-old man was referred to Foniatric department of ENT clinic, Clinical Centre Kragujevac, Serbia, for examination with the main complaints of hoarseness, dysphagia and exertional laborious breathing lasting for two months. We performed complete ENT examination and fiberoptic laryngoscopy revealed that the right side of the larynx was dislocated ventrally and left (Fig 1.). The airway was compromised and reduced to one-third but sufficient. Laryngeal videostroboscopyc findings show passive vertical right vocal folds vibration, left vocal folds vibration was normal. In exertional inhalation there was stridor. Pulmonologist exclude any respiratory problems so there was no indications for further functional respiratory diagnostics or examinations The routine biochemical tests were normal.

**Figure 2:**
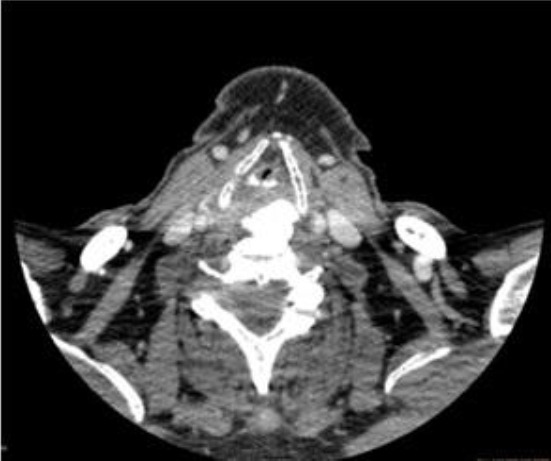
*Computed tomography of the neck shows elongated ossification of the frontal planes of the vertebral body especially at right side*.

Computed tomography of the neck (Fig. 2) revealed an elongated ossification of the frontal planes of the vertebral bodies, especially at the C 3-6 levels, with elongated and spiky spurs especially at the right side projecting into the soft tissues of the neck pushing hypopharynx, oesophagus and larynx ventrally and right. Computed tomography was also done with contrast to exclude possible malignant process. High density was not observed. Forestier’s disease was also confirmed by X-ray examination of column performed in two directions.

Patient was examined by orthopedic surgeon and his assessment was that there were no indications for surgical treatment. Orthopedic surgeon advised just physical therapy for our patient. We decided to treat conservatively our patient with nonsteroidal anti inflammatory drugs associated with intensive physical rehabilitation treatment. Physical therapy reduced pressure on larynx and nonsteroidal anti-inflammatory drugs probably decreased inflammation in that region. At the check up two months later, the clinical findings were better and the symptoms were reduced. At that time we performed multidimensional computerised voice analysis to quantify the voice characteristics of our patient. Acustic voice-signal data were measured for fundamental frequency (Fo): 268.08Hz elevation of Fo can be explained by increase of subglottic pressure, percent of jitter: 1.72% and percent of shimmer: 2.44%, SD: 8.16% and amplitude perturbation quotient: 1.94%. Multidimensional voice evaluation was done to make it possible to follow up improvement of voice in future. Vocal therapy was not conducted because there were no indications for vocal therapy

## Discussion

The characteristic traits of DISH are ossification of anterior ligaments and the anterior osteophyte bridges that appear at both sides and symmetrically, but in literature we find them described very rarely asymmetrically causing extraspinal symptoms such as laryngeal manifestation occurring with hoarseness, dysphagia and laborious breathing [5, 6].

The suggested pathogenesis of DISH indicates that ossification and new bone formation is the result of abnormal osteoblast cell growth/activity in the bony ligamentous region [7].

In our patient ossification and new bone formation were present at C3-6 pronounced on the left causing laryngeal manifestation. The possible mechanism is not only compression of the larynx and oesophagus but also paresis of terminal laryngeal nerve fibers, traumatic compromisation, and direct involvement of the cricoarytenoid joint [8]. Commonly appearing symptoms are dysphagia, which appears at about 3-20% of patients with DISH [8, 9] and airway symptoms where prevalence in patients with DISH was 11.2% [10].

Most patients with DISH can be managed conservatively, as we decided. Surgical excision is appropriate in patients with severe and progressive symptoms such as airway obstruction and progressive weight loss caused by dysphagia problems [5, 12, 13].

Surgical treatment may be followed by many complications. According to determination presence and impact of dysphonia and dysphagia following the anterior approach to the cervical spine in Winslow et al study, hoarseness was present in 51%, dysphagia was present in 60% while in 18% breathing difficulties were found postoperatively [13].

After appropriate history, clinical examination, diagnostic and radiological procedure we diagnosed Forestier’s disease as a cause of the symptoms in our patient. We treated the patient conservatively and he responded well to ehe therapy. In clinical practice it is advisable to take into consideration Forestier’s disease as a possible cause of hoarseness and dysphagia in rare cases.
